# Trend analysis and prediction of gonorrhea in mainland China based on a hybrid time series model

**DOI:** 10.1186/s12879-023-08969-4

**Published:** 2024-01-22

**Authors:** Zhende Wang, Yongbin Wang, Shengkui Zhang, Suzhen Wang, Zhen Xu, ZiJian Feng

**Affiliations:** 1 School of Public Health, Weifang Medical University, Weifang, China; 2 School of Public Health, Xinxiang Medical University, Xinxiang, China; 3 School of Basic Medicine, Institute of Basic Medical Sciences, Chinese Academy of Medical Sciences, Peking Union Medical College, Beijing, China; 4 Zibo Hospital of Shandong Health Group, Zibo, China; 5 Chinese Center for Disease Control and Prevention, Beijing, China; 6 Chinese Preventive Medicine Association, Beijing, China; 7 National Key Laboratory Of Intelligent Tracking And Forecasting For Infectious Diseases, Beijing, China

**Keywords:** Gonorrhea, Modeling, SARIMA, LSTM

## Abstract

**Background:**

Gonorrhea has long been a serious public health problem in mainland China that requires attention, modeling to describe and predict its prevalence patterns can help the government to develop more scientific interventions.

**Methods:**

Time series (TS) data of the gonorrhea incidence in China from January 2004 to August 2022 were collected, with the incidence data from September 2021 to August 2022 as the validation. The seasonal autoregressive integrated moving average (SARIMA) model, long short-term memory network (LSTM) model, and hybrid SARIMA-LSTM model were used to simulate the data respectively, the model performance were evaluated by calculating the mean absolute percentage error (MAPE), root mean square error (RMSE), and mean absolute error (MAE) of the training and validation sets of the models.

**Results:**

The Seasonal components after data decomposition showed an approximate bimodal distribution with a period of 12 months. The three models identified were SARIMA(1,1,1) (2,1,2)_12_, LSTM with 150 hidden units, and SARIMA-LSTM with 150 hidden units, the SARIMA-LSTM model fitted best in the training and validation sets, for the smallest MAPE, RMSE, and MPE.

**Conclusions:**

The overall incidence trend of gonorrhea in mainland China has been on the decline since 2004, with some periods exhibiting an upward trend. The incidence of gonorrhea displays a seasonal distribution, typically peaking in July and December each year. The SARIMA model, LSTM model, and SARIMA-LSTM model can all fit the monthly incidence time series data of gonorrhea in mainland China. However, in terms of predictive performance, the SARIMA-LSTM model outperforms the SARIMA and LSTM models, with the LSTM model surpassing the SARIMA model. This suggests that the SARIMA-LSTM model can serve as a preferred tool for time series analysis, providing evidence for the government to predict trends in gonorrhea incidence. The model's predictions indicate that the incidence of gonorrhea in mainland China will remain at a high level in 2024, necessitating that policymakers implement public health measures in advance to prevent the spread of the disease.

**Supplementary Information:**

The online version contains supplementary material available at 10.1186/s12879-023-08969-4.

## Introduction

In the year 2020, more than 1 million sexually transmitted infections (STIs) were acquired every day worldwide, about 22% of them were gonorrhea infections [1]. Gonorrhea is the trigger for many urinary diseases (e.g. pelvic inflammatory disease, ectopic pregnancy, maternal death maternal death, infertility, epididymitis, gonococcemia, and disseminated gonococcal infection) [2, 3]. In addition, it increases the risk of human immunodeficiency virus (HIV) acquisition [3].

In China, gonorrhea has become the second most prevalent STIs, after syphilis. Since the first gonorrhea case was reported in 1977 [4], it has been prevalent in China for 46 years and become a public health distress that requires attention. The gonorrhea incidence was 9.06 cases per 100,000 population in 2021 [5], there is an increase of 20.96% compared with the incidence in 2020 (7.49 cases per 100,000 population) [6]. Since the COVID-19 outbreak in 2019, the “zero-COVID” policy implemented by the Chinese government may have had an impact on the spread of many infectious diseases. With China's withdrawal from the “zero-COVID” policy at the end of 2022, the epidemiological trends of STIs will get more attention. Because of the characteristics of the long incubation period of STIs, the insidious nature of the transmission, and the difficulty of complete cure, the surveillance of STIs is challenging.

By analyzing the epidemiologic trends of disease, policymakers can develop response plans earlier and more accurately. The role of mathematical models is to quantify the internal patterns of TS data. Traditional ARIMA models were widely used in TS data analysis and forecasting, but one of the drawbacks is that it requires stationary data and does not fit well for non-linear TS data. Some studies have shown seasonal fluctuations in gonorrhea incidence [4, 7], the same pattern was found when we preprocessed the study samples, so a multiplicative seasonal ARIMA (SARIMA) model was needed. The machine learning (ML) theory and technology rapidly develop in the past several years. Similarly, ML was widely used to predict unknown TS data by analyzing historical data. Artificial neural networks (ANN) is one of the important algorithms for ML, ANN is a mathematical model with adaptive characteristics that simulate the structure and function of the biological neural network consisting of many neurons, which are interconnected by certain factors to form a powerful network for processing information. Theoretically, the ML can fit any kind of TS data with a very small error by iterating. The LSTM model was first proposed in 1997 as an extension to the ANN, which solved the problem that traditional ANN "forget" the initial input in continuous iterations owing to its special neuronal structures. As a typical representative of Recurrent Neural Networks(RNN), LSTM models have been proven to have good non-linear fitting capabilities. Due to the cell structure of LSTM, the LSTM model can store and access information over a long period of time when dealing with data with long time spans, thus alleviating the problem of gradient vanishing or explosion. Therefore, we attempt to use the LSTM method to establish a gonorrhea model. Since the hybrid ARIMA-ANN models can not only accurately track the stable long-term trends and seasonal characteristics of the original observed data, but also capture the nonlinear characteristics and stochastic fluctuations of the observations well, it often outperforms the single ARIMA model or ANN models in terms of simulation performance and prediction performance in practical applications, as demonstrated in many prediction studies of infectious diseases [8–10]. Therefore, we used the SARIMA, LSTM, and hybrid SARIMA-LSTM models to analyze the monthly time series data of gonorrhea infections in China from January 2004 to July 2022, and made predictions based on the model fitting results.

## Methods

### TS data collection

The monthly number of newly reported cases of gonorrhea from January 2004 to August 2020 was extracted from the reports of “*Overview of the national epidemic of notifiable infectious diseases*" published by the *Bureau for Disease Control and Prevention of China National Health Commission* every month (available from the website: http://www.nhc.gov.cn/jkj/new_index.shtml).

The data published by the government were extracted from the routine reporting system for notifiable infectious diseases covering 31 provinces in mainland China, which was established by the Chinese government in the 1950s and switched from paper-based reporting to web-based reporting in 2003 [11]. The case information of notifiable infectious diseases was timely reported from local hospitals and community health service centers throughout the country and was reviewed and confirmed by local *Centers for Disease Control and Prevention* (*CDC*) after confirmatory tests [11, 12]. The reports were updated to August 2022, therefore, data after this date are not available at present, a total of *N* = 224 observations spanning 18 years were included in the study.

### TS decomposition

TS decomposition means separating a TS into several distinct components, a deterministic and nonseasonal secular trend component (T_t_), a deterministic seasonal component with known periodicity (S_t_), and a stochastic irregular component (I_t_). After investigating the TS data by scrutiny of the recorded data plotted over time, we performed an additive decomposition of TS, which is expressed as *Yt* = *T*_*t*_ + *S*_*t*_ + *I*_*t*_*.* We confirmed the T_t_ by using a smooth weighted 13-term moving average filter given by:

$$\widehat{{y}_{t }}=\sum\limits _{j=-q}^{q}{k}_{j}\,{y}_{t+j}$$

*q* < *t* < *N – q,* and *q* = *6* for monthly data*,* because symmetric moving averages have an odd number of terms, a reasonable choice for the weights is* k*_*j*_ = *1/4q* for *j* =  ± *q,* and *k*_*j*_ = *1/2q* otherwise. By the transformation of TS, the first and last q observations were lost, so we repeated the first and last smoothed values six times. To calculate the S_t_*,* we used the seasonality moving average filter, expressed as:

$$\begin{array}{c}\widetilde{{s}_{k}}=\frac{1}{{n}_{k}}\sum\limits_{j=1}^{\left({}^{N}\!\left/ \!{}_{s}\right.\right)-1}{x}_{k+js}\\ \overline{s }=\frac{1}{S} \sum\limits_{k=1}^{s}\widetilde{{s}_{k}}\\ \widehat{{s}_{k}}=\widetilde{{s}_{k}}-\overline{s}\end{array}$$

For *s* = 12, *k* = 1,…,12, and ${\widetilde{{s}_{k}}}={\widetilde{s}}_{k-s}$ for *k* > *s.* Using $\widehat{{s}_{k}}$ to constrain the seasonality component to fluctuate around zero.

In time series trend analysis, the Mann–Kendall test is a widely used non-parametric test method for analyzing trend changes in a time series. The Mann–Kendall test does not require the sample to follow a certain distribution, is not affected by a few outliers. In a two-sided trend test with a specified test level of *α* = 0.05, the presence of a significant increasing or decreasing trend in the sequence can be inferred if the value of $\left|z\right|$> 1.96. A *z*-value greater than zero signifies an upward trend, while a *z*-value less than zero indicates a downward trend.

### Modeling of the SARIMA

#### Mathematical equations of the SARIMA model

The SARIMA model is always defined as SARIMA *(p, d, q) (P, D, Q)*_*s*_*,* where *p, d, and q* represent non-seasonal components, and *P, D, and Q* represent seasonal components. *p* and *P* are degrees of non-seasonal and seasonal autoregressive, respectively. *d and D* are degrees of non-seasonal and seasonal differencing, respectively. *q* and *Q* are degrees of the non-seasonal and seasonal moving average, respectively, and *s* denote the sampling period.

The SARIMA model polynomial with the lag operator can be expressed as:

$$\begin{array}{c}\varphi \left(L\right)\Phi \left(L\right)(1-{L)}^{d}{\left(1-{L}^{s}\right)}^{D}{y}_{t}=\theta \left(L\right)\Theta \left(L\right){\varepsilon }_{t}+constant\\ {L}^{i}{y}_{t}={y}_{t-i}\\ {\Delta }^{d}=(1-{L)}^{d}\\ {\Delta }_{s}=(1-{L}^{s})\\ \varphi \left(L\right)=1 - {\phi }_{1}L - \dots -{ \phi }_{p}{L}^{p}\\ \Phi \left(L\right)=1 - {\Phi }_{s}L - \dots -{ \phi }_{Ps}{L}^{Ps}\\ \theta \left(L\right)=1+ {\theta }_{1}L+ \dots +{ \theta }_{q}{L}^{q}\\ \Theta \left(L\right)=1+ {\Theta }_{s}L+ \dots +{ \Theta }_{Qs}{L}^{Qs}\end{array}$$

*ε*_*t*_ denotes a sequence of uncorrelated random variables from a defined probability distribution with a mean zero.

#### SARIMA model selection and parameters estimation

As long-term trends and seasonal fluctuations were observed in the TS, data transformations were necessary. After successively differencing to the TS, we tested the stability of the differenced TS (TS’) by an Augmented Dickey-Fuller (ADF) test. Then we conducted the Ljung-Box Q test on TS' to determine whether the sequence is a white noise sequence. The sample autocorrelation function (ACF) and partial autocorrelation function (PACF) are useful qualitative tools to assess the presence of autocorrelation at individual lags. The Ljung-Box Q-test is a more quantitative way to test for autocorrelation at multiple lags jointly. The null hypothesis for this test is that the first *m* autocorrelations are jointly *zero*. By plotting the ACF and PACF of TS’, we explored the lags of *p*, *q*, *P*, and *Q* of the SARIMA model, the best-fitting model was determined by minimizing Akaike information criteria (AIC) and Bayesian information criteria (BIC) among all reasonable model combinations. Basically, information criteria are likelihood-based measures of model fit that include a penalty for complexity (specifically, the number of parameters). Different information criteria are distinguished by the form of the penalty, and can prefer different models. Let log*L*($\widehat{\theta }$) denote the value of the maximized loglikelihood objective function for a model with *k* parameters fit to *N* data points. The AIC and BIC for a specific model are given by the formulas: -2log*L*($\widehat{\theta }$) + 2* k* and − 2log*L*($\widehat{\theta }$) + *k*log(*N*), respectively, The AIC compares models from the perspective of information entropy, as measured by Kullback–Leibler divergence. The BIC compares models from the perspective of decision theory, as measured by expected loss. In comparing AIC and BIC values among multiple models, lower criterion values are preferred.Subsequently, a method of maximum likelihood is employed to estimate the parameters of the model. The statistical significance of a parameter is ascertained based on the *t*-test statistic and the corresponding *p*-value of each parameter.

#### Goodness-of-fit checks of the SARIMA model

The mean absolute percentage error (MAPE), root mean square error (RMSE), and mean absolute error (MAE) were used as indicators for evaluating the goodness-of-fit of the model, which were given by:

$$\begin{array}{c}MAPE=\frac{100\%}{N} \sum\limits_{t=1}^{N} \frac{\left|{x}_{t}-{y}_{t}\right|}{{x}_{t}}\\ RMSE=\sqrt{\frac{1}{N} \sum\limits_{t=1}^{N}{ \left({x}_{t}-{y}_{t}\right)}^{2}}\\ MAE= \frac{1}{N} \sum\limits_{t=1}^{N} \left|{x}_{t}-{y}_{t}\right|\end{array}$$

Where *x*_*t*_ and *y*_*t*_ denote the observation series and fitting series, respectively. These three metrics are commonly used to assess the goodness-of-fit of models, reflecting the discrepancy between the actual and predicted values. Therefore, the smaller the values of these three indicators, the smaller the error in the model fit. Specifically, MAPE represents the percentage of the difference between the fitted value and the actual value relative to the actual value. This not only considers the difference between the fitted value and the actual value but also takes into account the ratio of the difference to the actual value, thus enabling an assessment of the quality of the model. The advantage of the RMSE metric is that its value is consistent with the order of magnitude of the original data, and its interpretation can be expressed as the average difference between the fitted value and the actual value. MAE denotes the mean absolute deviation between the fitted and actual values, which also reflects the fitting effect of the model.

We conducted a Ljung-Box Q-test, along with the ACF and PACF plots on the residual series to check the autocorrelation. Finally, we performed normality diagnostics by plotting the histogram of standard residuals and Quantile–Quantile (QQ) plot of residuals.

**Fig. 1:**
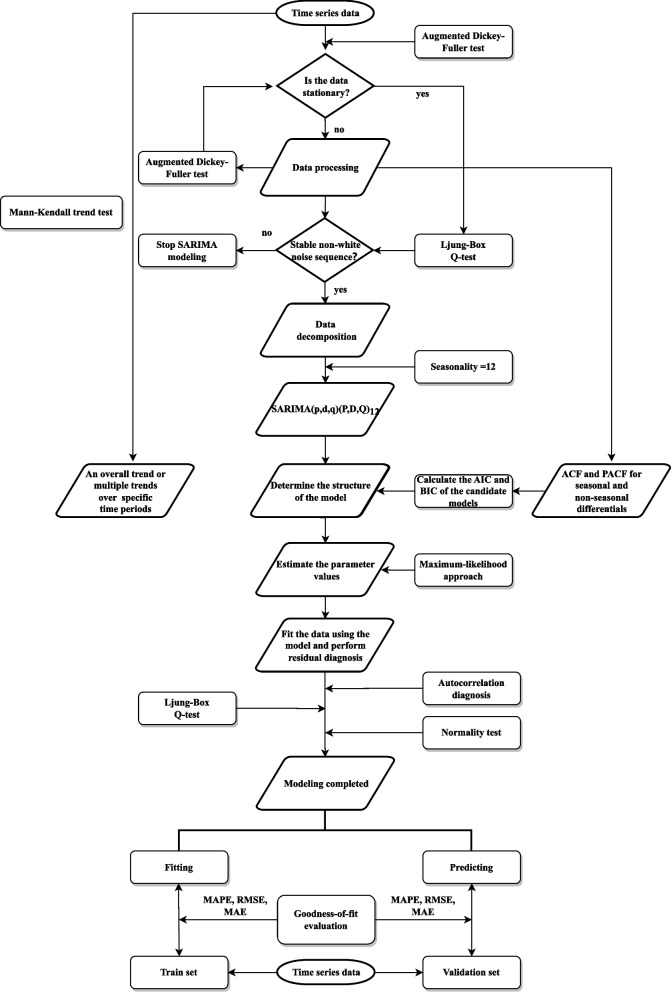
Flowchart of SARIMA model construction and simulation

To test the effectiveness of the model prediction, we used the method of setting up a training set and a test set to verify the prediction accuracy. We used the last 12 months’ (September 2021 to August 2022) data of the time series as the validation, using the data before September 2021 for modeling, and then performing the prediction with a time step of 12, and calculating the goodness-of-fit evaluation indicators for the validation set and the training set, respectively. The process of constructing and simulating the SARIMA model is shown in Fig. 1.

### Modeling of the LSTM

#### LSTM network architecture

**Fig. 2:**
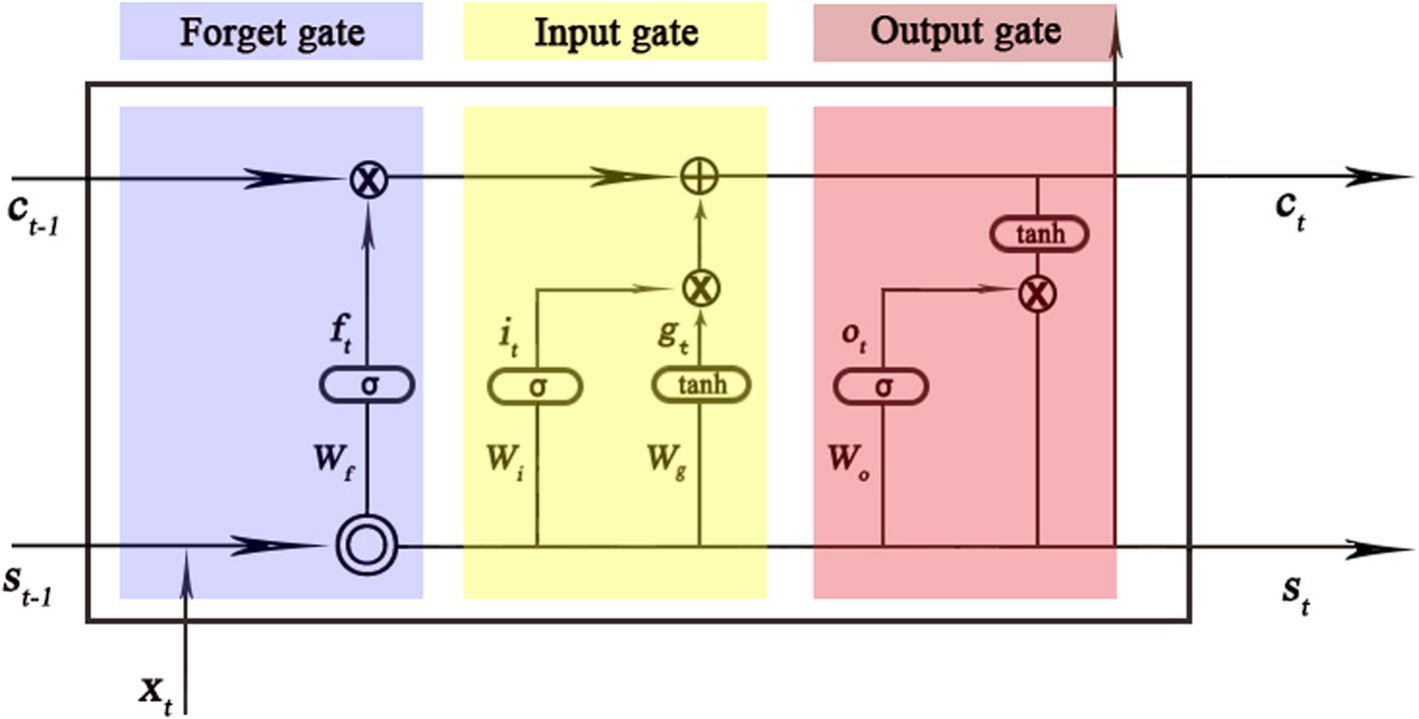
The cell structure of LSTM. The arrow indicates the data flow, where *x*, *s*, *c*, *f*,* i*, *g*, and *o* denote the input, output, cell state, forget gate, input gate, cell candidate, and output gate in time step *t*, respectively. *σ* and *tanh* denote the *sigmoid* activation function and the hyperbolic tangent function, which maps the data to (0,1) and (-1,1), respectively. $\otimes$, $\oplus$ are vector operators which represent element-wise multiplication and element-wise addition, respectively

LSTM is a kind of recurrent neural network (RNN) with a special structure [13]. An LSTM network consists of a sequence input layer, an LSTM layer, and an output layer. Different from the traditional RNN, there is a cell state in the LSTM neurons, which can effectively retain long-term memory and solve the problem of gradient disappearance. The cell state contains information learned from the previous time steps. At each time step, the layer adds information to or removes information from the cell state, all these updates are controlled by *gates.* There are three kinds of gates in the LSTM layer, input gate (*i*), forget gate (*f*), and output gate (*o*), Fig. 1 illustrates the flow of data at time step *t* and shows how the *gates* forget, update, and output the cell and hidden states. The cell structure of LSTM are shown in Fig. 2.

The following formulas describe the components at time step *t*:

$$\begin{array}{c}{f}_{t}=\sigma \left({W}_{f}\cdot \left[{S}_{t-1},{ X}_{t} \right]+{b}_{f}\right)\\ {i}_{t}=\sigma \left({W}_{i}\cdot \left[{S}_{t-1}, {X}_{t} \right]+{b}_{i}\right)\\ {g}_{t}=tanh\left({W}_{g}\cdot \left[{S}_{t-1},{ X}_{t} \right]+{b}_{g}\right)\\ {o}_{t}=\sigma \left( {W}_{o}\cdot \left[{S}_{t-1}, {X}_{t} \right]+{b}_{o}\right)\\ {C}_{t}={f}_{t}\otimes {C}_{t-1}+ {i}_{t}\otimes {g}_{t}\\ {S}_{t}={O}_{t}\otimes tanh({C}_{t})\end{array}$$

Where *W*,* b* denote the matrices of input weight and bias, respectively.

#### TS data normalization

Data normalization can improve the training efficiency and generalization ability of the model, and accelerate the speed of gradient descent to obtain the optimal solution. A *Z-Score* method was used to normalize the sample data, which was given by: TS* = (*TS − μ*) */ σ,* where *μ* and* σ* denote the sample mean and standard deviation.

#### Define LSTM network architecture and training

To prevent the gradients from exploding, set the gradient threshold to 1. The number of hidden units, the times of maximum iterations, and the learning rate of the LSTM network both influenced the fitting accuracy of the models. To prevent overfitting or underfitting, under the condition that the initial learning rate was set to 0.005, we have experimented with various combinations of the number of hidden units, and the number of maximum iterations, and took the RMSE as the indicator to evaluate the fitting accuracy. To automatically drop the learning rate during training, using a piecewise learn rate schedule, multiply the initial learning rate by a drop factor of 0.2 after half of the maximum iterations. We used the "Adam" solver to update the network parameters by taking small steps in the direction of the negative gradient of the loss function. The solvers update the parameters using a subset of the data at each step, each parameter update is called an iteration. The current mainstream method for determining the LSTM structure is trial-by-error. We establish the optimal model structure based on the similarity degree of the time series plots of the fitted and actual values, along with the RMSE values. We conduct experiments using 10, 50, 100, 150, and 200 hidden neurons respectively, executing iterations in increments of 50 from 50 to 500. During this process, we calculate the RMSE of the model fit for each combination and perform three training runs for each combination to compute the average RMSE value. The criterion for selecting the model structure is the minimum value of the average RMSE.

#### Goodness-of-fit checks of the LSTM model

As mentioned before, to ensure the accuracy of the model fit, we trained the model using the training set and validate the accuracy of the model prediction using the validation set. We calculated the MAPE, RMSE, and MAE separately for the training and validation sets for evaluating the goodness-of-fit of the model, which were shown in Goodness-of-fit checks of the SARIMA model.

#### Forecasting future time steps

To forecast the data of multiple time steps in the future, predict time steps one at a time and update the network state at each prediction. For each prediction, use the previous prediction as input to the function. To forecast the values of future time steps of a sequence, specify the responses to be the training sequences with values shifted by one-time step. That is, at each time step of the input sequence, the LSTM network learns to predict the value of the next time step [14]^.^ The predictors are the training sequences without the final time step. So the data from January 2004 to July 2022 of TS* were divided into the input sequence, and the data from February 2004 to August 2022 of TS* were divided into the output sequence.

### Construction of the hybrid SARIMA-LSTM model

The SARIMA model can fit seasonal fluctuations well, but the fitting accuracy is poor for nonlinear components of TS data, while the LSTM model can compensate for this deficiency well. Since real-time series data might not have a strict cyclical fluctuation pattern, another problem is that the mandatory fitting of seasonal fluctuations using a single LSTM model over a longer period increases the risk of overfitting, then combining the two models into a hybrid SARIMA-LSTM model can solve the accuracy problem of nonlinear fitting and simulate seasonal fluctuations at the same time. The route of designing the SARIMA-LSTM model is to use the fitting result of the SARIMA model as the input of LSTM and real TS data as the output, simulate the output series of the SARIMA model using the architecture and parameters of the LSTM model, and then update the network with real TS data. The goodness-of-fit test and implementation of the prediction of the SARIMA-LSTM model has shown in the Goodness-of-fit checks of the SARIMA model and Goodness-of-fit checks of the LSTM model.

### Softwares and significant level

Matlab R2020a (MathWorks Corporation) was used to perform the models involved in the study, and Microsoft Office 2013 (Microsoft Corporation) for data collection and processing. Statistical significance level was set at a two-sided *p* < 0.05. In statistics, the *p*-value is a measure used to evaluate the strength of evidence against a null hypothesis. It represents the probability of observing a test statistic as extreme or more extreme than the one actually observed, assuming that the null hypothesis is true.

## Results

### Trends and seasonality of the TS

**Fig. 3:**
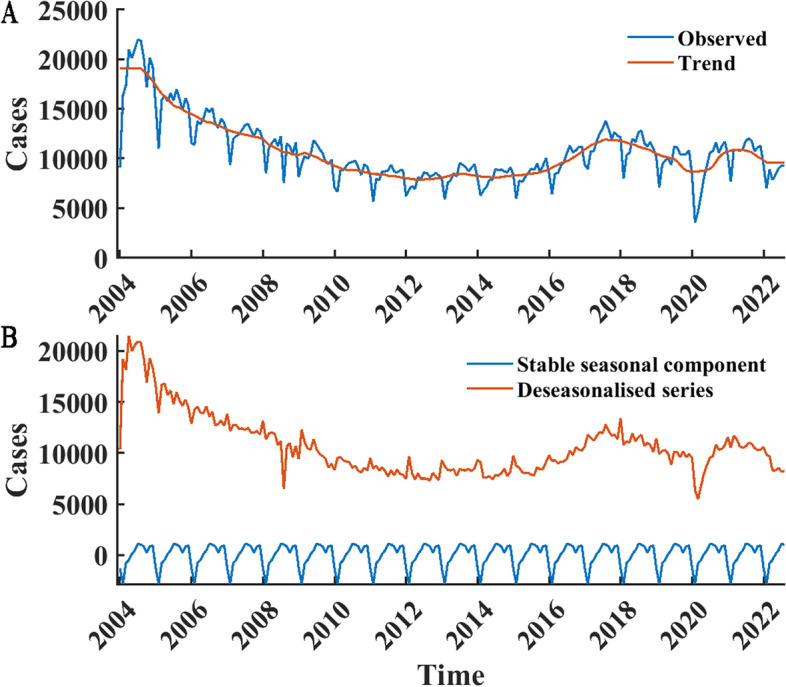
Monthly TS data of gonorrhea infections from JAN 2004 to AUG 2022 and the TS decomposition. The blue curve in **A** represents the incidence time series, the red curve represents the long-term trend, the red curve in **B** represents the time series without the seasonal component (both long-term trend and stochastic component exist), and the blue curve in **B** represents the stable seasonal component with periodicity 12

From January 2004 to August 2022, the average number of cases per month was 10,683. The maximum and minimum monthly incidence occurred in July 2004 and February 2020, with 21, 961 and 3, 524 cases, respectively. As shown in Fig. 3A, The long-term trend of gonorrhea infections can be approximated as a combination of the alphabets "u" and "m". Upon conducting the Mann–Kendall trend analysis, the trend test *z*-values for the number of cases were found to be -11.636, 4.959, -3.496, and 0.142 for the periods of January 2004 to February 2015, March 2015 to August 2017, September 2017 to February 2020, and March 2020 to August 2022, respectively. Based on the test level of α = 0.05, it can be inferred that the trend in the incidence of gonorrhea cases exhibits an initial decline, followed by an increase, and subsequently another decline. Over the entire research period, the trend test *z*-value is -5.772, signifying a general decrease in the overall trend.In detail, from January 2004 to August 2017, the trend shows a decline followed by an increase, from September 2017 to February 2022, it shows a similar trend. By decomposing the data, a periodicity of 12 was found, and the stable seasonal component curves showed a bimodal distribution of the number of cases within a period, with peak months of July and December, respectively (Fig. 3B).

### SARIMA model selection

**Fig. 4:**
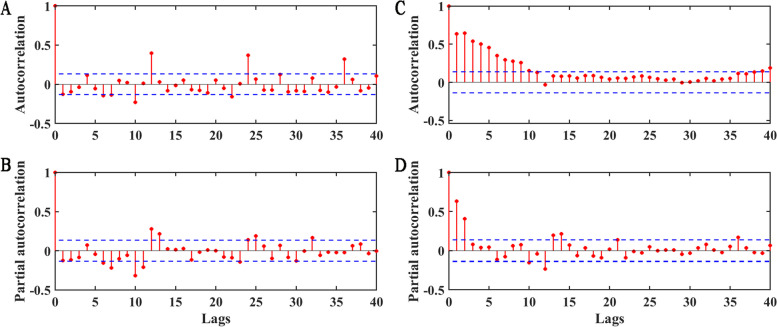
ACF and PACF of the differenced TS. **A** and **B** denote the ACF and PACF of the non-seasonal differential series. **C** and **D** denote the ACF and PACF of the seasonal differential series, respectively. The red stem plots represent the sample ACF and PACF values at different lags, and the blue dashed lines indicate the ± 2 times standard deviation interval

We performed the ADF test on the differentially transformed TS data, and the results showed that the differential series were stationary (*t* = -17.312,* p* < 0.05). According to the ACF and PACF plots, the order of *p, q* was temporarily set as 1 or 2, and *P* was set as 2 (Fig. 4). By comparing the AIC and BIC of all alternative models (Table 1), the SARIMA model was finally determined as SARIMA (1, 1, 1) (2, 1, 2) _12_. The constant was not included, because the hypothesis test results for the constant were not statistically significant (*t* = 0.086, *p* = 0.931). The model can be expressed as a polynomial of:

**Table 1:** Alternative SARIMA models and the AIC and BIC values

ID	Models (S = 12)	AIC	BIC
1	SARIMA(1,1,1) (2,1,0)	3591.00	3606.80
2	SARIMA(1,1,1) (2,1,1)	3563.80	3582.80
**3**	**SARIMA(1,1,1) (2,1,2)**	**3543.80**	**3569.00**
4	SARIMA(1,1,2) (2,1,0)	3582.00	3600.90
5	SARIMA(1,1,2) (2,1,1)	3562.10	3584.20
6	SARIMA(1,1,2) (2,1,2)	3563.80	3582.80
7	SARIMA(2,1,1) (2,1,0)	3581.40	3600.30
8	SARIMA(2,1,1) (2,1,1)	3562.80	3584.90
9	SARIMA(2,1,1) (2,1,2)	3563.60	3588.80
10	SARIMA(2,1,2) (2,1,0)	3582.80	3604.90
11	SARIMA(2,1,2) (2,1,1)	3562.30	3587.60
12	SARIMA(2,1,2) (2,1,2)	3545.30	3573.70

$$(1-{\varphi }_{1} L)(1-{\Phi }_{12}{L}^{12}-{\Phi }_{24}{L}^{24})\left(1-L\right)\left(1-{L}^{12}\right){y}_{t}=\left(1+{\theta }_{1} L\right)\left(1+{\Theta }_{12}{L}^{12}+{\Theta }_{24}{L}^{24}\right){\varepsilon }_{t}$$

Where the results of the estimation of the parameters were shown in Table 2.

**Table 2:** SARIMA(1, 1, 1) (2, 1, 2)_12_ parameters estimation

Parameters	Value	Standard error	*t*-statistic	*p*
*φ*_*1*_	-0.251	0.087	-2.882	0.004*
*Φ*_*12*_	-1.000	0.057	-17.462	< 0.001*
*Φ*_*24*_	-0.215	0.059	-3.626	< 0.001*
*θ*_*1*_	-0.218	0.101	-2.165	0.030*
*Θ*_*12*_	0.297	0.090	3.300	0.001*
*Θ*_*24*_	-0.448	0.087	-5.116	< 0.001*

^*^Under the premise of a test level α = 0.05, the hypothesis test of the parameters is statistically significant

### Selection of LSTM parameter combinations

**Fig. 5:**
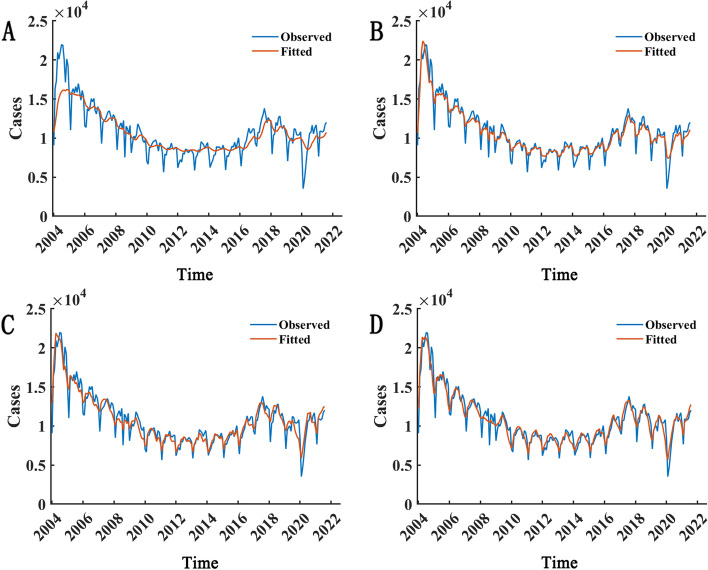
Comparison of fitted values from various underfitted LSTM models with actual data. **A**-**D** correspond to scenarios of 10 hidden units with 50 iterations, 10 hidden units with 500 iterations, 50 hidden units with 50 iterations, and 50 hidden units with 500 iterations, respectively. Here, the blue curve signifies actual incidence data, while the red curve denotes LSTM model-fitted data

In order to identify the optimal parameter combination, we tested various combinations of hidden units and iteration numbers, calculating the goodness of fit (represented by RMSE) for the LSTM model under different conditions. We initially attempted to use 10 neurons for 50 iterations, but the model's fitted values did not align well with the actual data curve(Fig. 5). Consequently, we increased the number of iterations to 100, but the model still failed to fit the data adequately. We then tested 150, 200, 250, 300, 350, 400, 450, and 500 iterations, but the fitting curves remained unsatisfactory despite the model's low RMSE values. We subsequently increased the number of neurons to 50 and performed 50, …, 500 iterations, yielding results similar to those of the model with 10 neurons. Therefore, we experimented with 150 and 200 neurons for 50, …, 500 iterations and calculated the goodness of fit for each model.

Through various parameter combinations and multiple training and validation sessions, we ultimately determined that the optimal number of hidden units was 150, with a maximum of 150 iterations, as shown in Table 3. When the number of hidden units was 10 or 50, or the number of iterations was less than 50, we observed underfitting in the model fitting curves, so we did not proceed to second and third training rounds. However, when the number of iterations exceeded 300, overfitting typically occurred, as evidenced by the increased RMSE values. To minimize the impact of random factors, we trained the candidate models three times each, calculating the average RMSE value for each model and selecting the parameter combination with the smallest average RMSE for model establishment. As LSTM iterations yield different correction values, the chosen parameter combination should provide a general representation of the model's fitting capabilities. Furthermore, considering time cost and computational power, we abandoned the approach of using fewer neurons with more iterations when a greater number of neurons and fewer iterations could accurately fit the data.

**Table 3:** LSTM model fitting accuracy for different combinations of hidden units and iterations

ID	Num Hidden Units	Iterations	RMSE1	RMSE2	RMSE3	MRMSE^a^
1	10	50	1400.40	-	-	-
2	10	100	781.27	-	-	-
3	10	150	805.84	-	-	-
4	10	200	788.77	-	-	-
5	10	250	840.84	-	-	-
6	10	300	875.89	-	-	-
7	10	350	860.04	-	-	-
8	10	400	920.32	-	-	-
9	10	450	1038.70	-	-	-
10	10	500	880.61	-	-	-
11	50	50	738.01	-	-	-
12	50	100	824.52	-	-	-
13	50	150	936.04	-	-	-
14	50	200	973.73	-	-	-
15	50	250	1015.70	-	-	-
16	50	300	1119.60	-	-	-
17	50	350	1260.40	-	-	-
18	50	400	1257.50	-	-	-
19	50	450	1242.40	-	-	-
20	50	500	1422.60	-	-	-
21	100	50	839.63	-	-	-
22	100	100	879.49	1301.90	1283.10	1154.30
23	100	150	1141.20	1160.10	1135.80	1145.70
24	100	200	1279.20	1080.10	1231.60	1196.97
25	100	250	1295.20	1359.20	1378.90	1344.43
26	100	300	1359.10	1287.00	1378.90	1341.67
27	100	350	1307.40	1329.40	1436.50	1357.77
28	100	400	1321.50	1421.30	1373.90	1372.23
29	100	450	1395.20	1448.60	1396.90	1413.57
30	100	500	1455.70	1445.20	1451.20	1450.70
31	150	50	895.45	-	-	-
32	150	100	1121.00	1102.90	2036.70	1419.66
**33**	**150**	**150**	**1090.00**	**1269.60**	**950.66**	**1103.42**
34	150	200	1200.80	1287.50	1381.20	1289.83
35	150	250	1279.80	1253.80	1359.20	1297.60
36	150	300	1326.80	1288.90	1383.80	1333.17
37	150	350	1349.20	1432.50	1412.10	1397.93
38	150	400	1401.30	1454.00	1409.00	1421.43
39	150	450	1436.00	1402.30	1388.10	1408.80
40	150	500	1425.50	1439.00	1433.40	1432.63
41	200	50	972.46	-	-	-
42	200	100	1125.70	1710.90	845.80	1226.66
43	200	150	1307.50	1175.60	1069.30	1184.13
44	200	200	1320.70	1298.30	1353.20	1324.07
45	200	250	1345.30	1363.00	1371.50	1359.93
46	200	300	1400.70	1353.10	1400.90	1384.90
47	200	350	1335.00	1390.00	1396.90	1373.97
48	200	400	1361.70	1346.20	1440.10	1382.67
49	200	450	1410.80	1448.90	1426.80	1428.83
50	200	500	1477.70	1431.10	1480.90	1463.23

^a^denotes the average of RMSE1, RMSE2, RMSE3. – means the training process was not implemented due to underfitting

### Residuals diagnostics of SARIMA model

We performed the Ljung-Box Q-Test on the residual series, and the test results (χ^2^ = 23.156, *p* = 0.281) indicates that the residual series is stationary and does not exhibit autocorrelation. However, between 2004 and 2009, the oscillation of the residuals fitted by the three models around *zero* is larger compared to other periods (Fig. 7).

The standardized residuals were obtained by normalized transformation of the residuals, and the histogram of the frequency distribution of the standardized residuals was plotted, and the results show that the frequency plot of the standardized residuals indicates a *zero*-centered symmetric, approximately normal distribution. The residuals QQ plots also show similar characteristics.

**Fig. 6:**
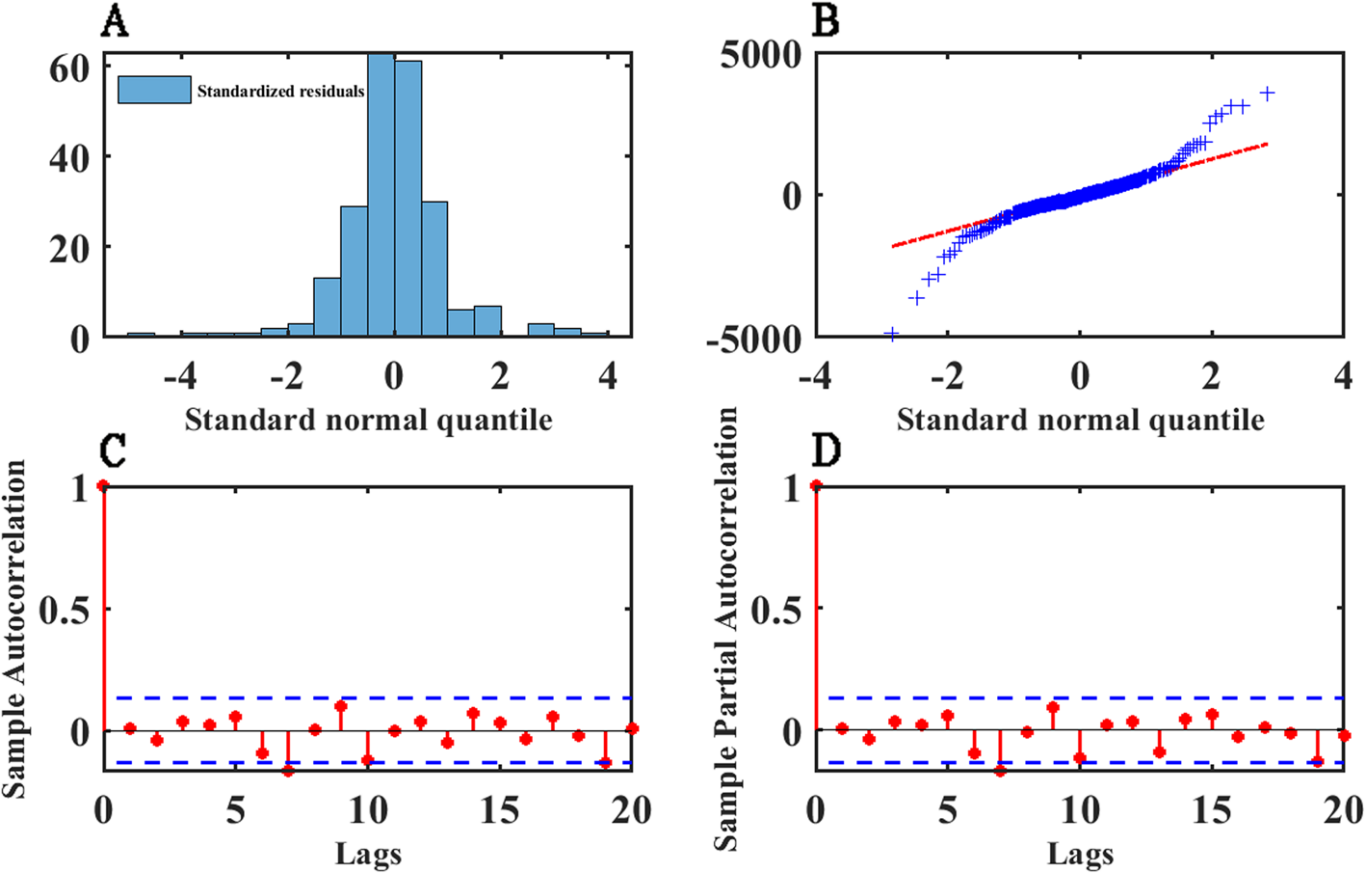
SARIMA model residuals normality and autocorrelation diagnostics. **A** shows the frequency distribution of standardized residuals using a histogram. **B** is the QQ plots of residuals of the SARIMA model, and the red dashed line represents the standard normal distribution. **C** and **D** is ACF and PACF of residuals, respectively. The stem plots represent the sample ACF and PACF values at different lags, and the blue dashed lines indicate the ± 2 times standard deviation interval

The residuals ACF and PACF plots show that most of the residuals are within the ± 2 standard deviation interval, but the residuals ACF and PACF exhibit significant autocorrelation at lag 7 (Fig. 6).

### Comparison of SARIMA, LSTM, and SARIMA-LSTM model fitting

**Fig. 7:**
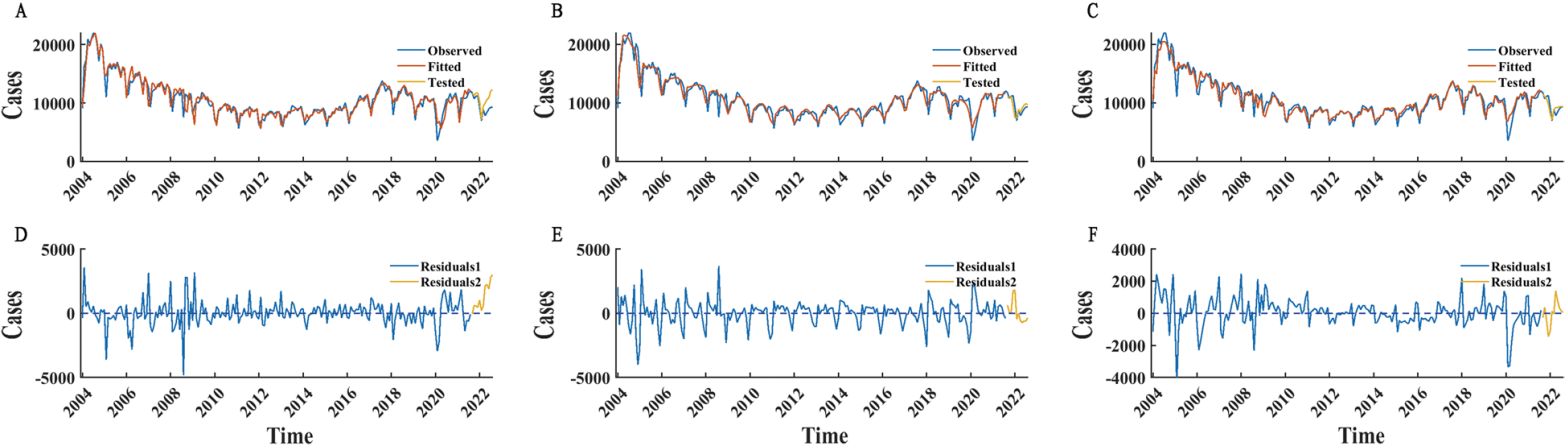
TS data fitting and validation by using SARIMA, LSTM, and SARIMA-LSTM models. In Fig. 7, the blue curves depicted in panels **A**, **B **and C represent the actual number of cases in China from January 2004 to August 2021. The red curves in panels **A**, **B**, and **C** correspond to the cases fitted by the SARIMA, LSTM, and SARIMA-LSTM models, respectively. The yellow curves in panels **A**, **B **and **C** correspond to the cases predicted by the SARIMA, LSTM, and SARIMA-LSTM models, respectively. Panels **D**, **E **and **F** display the simulation and prediction residuals for the SARIMA, LSTM, and SARIMA-LSTM models, represented by the blue and yellow curves, respectively

The SARIMA, LSTM, and SARIMA-LSTM models are used to fit the sample data, and the fitting plots are shown in Fig. 7. The goodness-of-fit of the SARIMA, LSTM, and SARIMA-LSTM models for the TS data can be assessed based on the values of MAPE, RMSE, and MAE, as shown in Table 4. Among the three models, the SARIMA-LSTM model demonstrates a superior overall fit, with MAPE, RMSE, and MAE values of 7.10%, 900.237, and 626.965, respectively. Furthermore, the predictive performance of the SARIMA-LSTM model surpasses that of the other two models, exhibiting MAPE, RMSE, and MAE values of 5.86%, 737.967, and 546.297, respectively, for the validation set.

**Table 4:** Evaluation of goodness-of-fit of SARIMA, LSTM, and SARIMA-LSTM models

Models	Fitting			Validation		
	MAPE(%)	RMSE	MAE	MAPE(%)	RMSE	MAE
SARIMA	6.97%	995.056	667.858	14.38%	1635.702	1290.466
LSTM	7.10%	992.593	709.762	6.77%	848.536	636.020
SARIMA-LSTM	7.10%	903.074	626.965	5.86%	737.967	546.297

### Forecasting with SARIMA, LSTM, and SARIMA-LSTM models

**Fig. 8:**
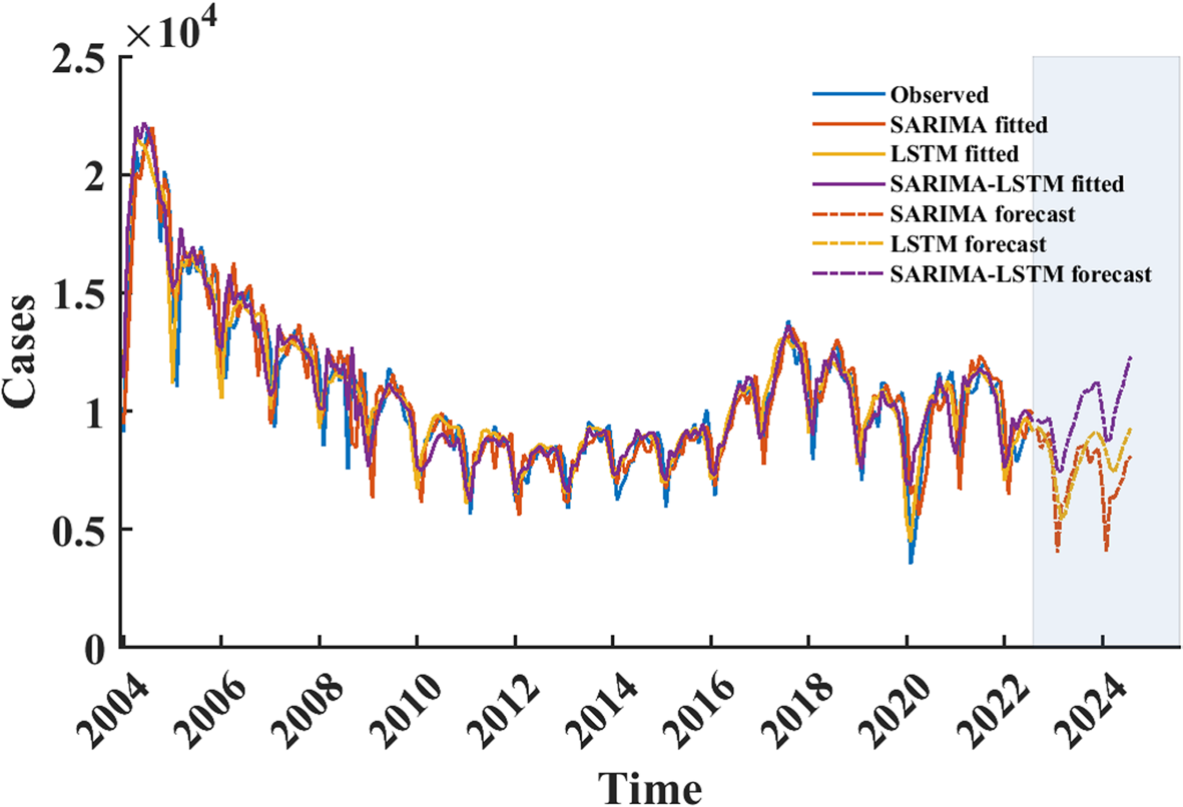
Prediction results from September 2022 to August 2024 of SARIMA, LSTM, and SARIMA-LSTM models. The light blue area represents the forecast period, and the red, yellow, and purple dashed curves indicate the prediction results of SARIMA, LSTM, and SARIMA-LSTM models, respectively. The red, yellow, and purple curves indicate the simulating results of the updated SARIMA, LSTM, and SARIMA-LSTM models using all observed data

We updated the SARIMA, LSTM, and SARIMA-LSTM models with all time series data before predicting future time steps to ensure the accuracy of the predictions. We used the updated SARIMA, LSTM, and SARIMA-LSTM models to predict the monthly incidence of gonorrhea for the next 24 months (September 2022 to August 2024), the results showed that the number of monthly incidences and trends predicted by SARIMA and LSTM model were relatively similar, and the number of monthly incidences did not exceed 10,000 during the prediction period. In each month of the forecast period, the predictions of the SARIMA-LSTM model are larger than those of the other two models. The results of the three models are similar for trend estimates, with a peak incidence in the winter of 2023 (Fig. 8).

## Discussion

According to our results, there are 118.9 million new gonorrhea infections annually from 2019 to 2021 in mainland China, 44.2% higher than the world level, according to the WHO reports, there are approximately 82.4 million new gonorrhea infections annually [15]. We observed a sharp decline in the number of reported gonorrhea cases in early 2020, especially in February 2020, so the models we used were not successful in fitting the data at this point. Similar characteristics were observed for several notifiable STIs other than gonorrhea, such as AIDS and syphilis [12], probably because the Chinese government took the strategy of lockdown to response the COVID-19 outbreak, the movement of the population was strictly controlled, most social activities in China have almost come to a standstill, and it was the Chinese New Year, when most public health practitioners were on vacation, resulting in a temporary delay in the surveillance and detection.

The seasonality of gonorrhea was first reported in the United States in 1971, with peaks in the summer and early autumn [7], and our seasonal diagnosis showed that the rates of gonorrhea had two peaks in the year, on average, in July and December, which is similar to the results of another study [16]. One study showed the risk of gonorrhea infection was highest at the temperature range of 6–11 °C [4], as the meteorological factors contribute to less than 20% [4] of the variation in infection transmission, so another possible reason for the seasonal fluctuations in gonorrhea is the frequency of sexual activity, which is consistent with the changing patterns of sexually transmitted diseases in the population [17].

SARIMA and ANN models have been successfully used to fit and predict time series data in a variety of fields [18–22]. The SARIMA model can fit seasonal fluctuations well, but the fitting accuracy is poor for nonlinear components of TS data [23], while the LSTM model can compensate for this deficiency well, but another problem is that the mandatory fitting of seasonal fluctuations using a single LSTM model over a longer period increases the risk of overfitting, so a hybrid SARIMA-LSTM model was used to solve the accuracy problem of nonlinear fitting and simulate seasonal fluctuations at the same time [24]. In the construction of the LSTM model, we initially utilized 10 hidden units and performed iterations from 50 to 500. Despite the low RMSE value, the curve fitting was suboptimal, leading us to hypothesize that the limited number of neurons restricted the model's ability to learn the seasonal variations of the original data. Consequently, we trialed 100 neurons, and found that with over 150 iterations, the model could effectively fit the original data. However, as the number of iterations exceeded 150, the RMSE value consistently increased, implying a boundary at 150 iterations and suggesting that 100 neurons were sufficient for learning the data's hidden features. We then tested the model with 150 and 200 neurons, and concluded that the LSTM model could capture the original data information with 150 iterations, thus rendering further iterations unnecessary. The RMSE value also supported this finding, as it did not significantly differ between 150 and 200 iterations but increased gradually afterwards, indicating an overfitting phenomenon. In summary, we determined that an LSTM model with 100 or 150 neurons and 150 or 200 iterations could effectively capture the information in the original data. We repeated the process three times and selected the model with the smallest average RMSE value, which consisted of 150 neurons and 150 iterations. Despite the theoretical possibility of achieving the same results with fewer hidden neurons and more iterations, employing more neurons allowed for quicker information capture, eliminating the need for redundant iterations and saving both time and computational resources. The fitting results of the three models also illustrate this point, and the SARIMA-LSTM model has the best goodness-of-fit, which indicates a good combination of the advantages of the other two models. When using the validation set to test the predictive performance of the models, the goodness-of-fit evaluation metrics in the validation set showed that the SARIMA model did not meet expectations, though the SARIMA model performed similarly to other models in the training set. And the SARIMA-LSTM model outperforms the LSTM model. In terms of predictive power, predictive models are considered perfect when the MAPE value is less than 5%. Models with MAPE values in the range of 5%-10% are considered high-precision models; models with MAPE values in the range of 10%-20% are considered good models [25]. In the validation set, both the LSTM and SARIMA-LSTM models have MAPE values between 5 and 10%, meeting the criteria for high-precision models, while the SARIMA model is only considered a good model, as its goodness-of-fit metric MAPE value is larger than 10%. The SARIMA-LSTM model's RMSE value for predicting the validation set is 737.967, which can be interpreted as the average difference between the predicted value and the actual value being 737.967, a reduction of 54.88% and 13.03% compared to the SARIMA and LSTM models, respectively. The magnitude of the MAE value reflects that the average absolute error of the SARIMA-LSTM model is 57.66% and 14.10% lower than that of the SARIMA and LSTM models, respectively. The advantage of the hybrid SARIMA-LSTM model is that it is more accurate for both long-term trends and stochastic components, because one of the steps of SARIMA-LSTM modeling is to simulate the output values of the SARIMA model and to be able to correct the model parameters through continuous iterations to reduce the error, the output of the SARIMA model has less variance compared to the original observations, so the learning cost of the SARIMA-LSTM model is smaller than that of the LSTM, and the fitted results will be more stable [26]. In other words, the SARIMA-LSTM model fits a more regular time series, thereby mitigating the impact of random fluctuations present in the original time series on the results. While our time series sample contains seasonality, it is not strictly seasonal due to the inclusion of non-linear random fluctuations. The SARIMA model can simplify this pattern into a pure seasonal fluctuation, a task that the LSTM model is incapable of, which may account for the latter's subpar fit to the seasonal regularities in the original data. The SARIMA-LSTM model effectively overcomes this shortcoming, thus exhibiting superior goodness of fit and predictive performance.

The SARIMA and LSTM models have similar goodness-of-fit evaluation metrics, and the prediction results are close, the SARIMA-LSTM model has larger prediction results than the other two models, the reason may be the SARIMA-LSTM model is a SARIMA model nested in the LSTM model, and its neuron structure is the same as that of the LSTM model, and the prediction process is carried out in the same way as the LSTM model, both using the previous value to predict the next predicted one [23], so the predicted value relies heavily on the fitted values. Moreover, we discovered that during January and February 2020, the fitted values of the SARIMA-LSTM model were higher than those of the other two models. This period saw actual data being potentially smaller due to lockdown measures implemented in China, which means that the SARIMA-LSTM model was closer to reality. This phenomenon reflects the greater flexibility of the hybrid SARIMA-LSTM model. However, considering that China has adjusted its “Zero-COVID” strategy at the end of 2022 by adopting only non-pharmaceutical interventions such as wearing masks and no more lockdowns, which will lead to an increase in population movement, the intensity of gonorrhea prevalence may increase as a result.In 2009, the Chinese government launched a new round of healthcare reform, and the “*National Basic Public Health Service Program*” was published, meanwhile, reporting and handling of infectious diseases as well as health supervision is also included [27]. The Chinese government has increased education on STIs, including gonorrhea, and people are paying more attention to the prevention of these diseases and have more access to knowledge about them, which has slowed down the spread of gonorrhea to some extent. But apparently, government propaganda is not enough, because sex is a taboo subject in traditional Chinese culture, and people rarely talk about it publicly. Parents rarely give their children the right advice in this regard. Sex education in schools is also not adequate for primary and secondary schools, leading to a lack of or even wrong perceptions of sexuality among young people. Timely detection and effective treatment can prevent further transmission of gonorrhea. However, the availability of effective treatment is being threatened as gonorrhea bacteria have successively acquired resistance to each of the antimicrobial agents used for treatment [28]. Emerging resistance to cephalosporins and macrolides and a dwindling pipeline of antimicrobial drug development threaten to undermine gonorrhea control and pose an even greater management challenge.

Admittedly, this study has several limitations. First, although the sample data were acquired from the official health administration in China, they were reported and aggregated by regional healthcare institutions at all levels, and between December 2019 and December 2022, the Chinese government has taken strict public health measures in response to the COVID-19 outbreaks, which could lead to a decrease in the willingness of gonorrhea patients to seek medical care and reduced access to treatment, so the data may be subject to reporting bias. Second, although the LSTM model has high fitting accuracy, the training progress and parameter optimization of the model requires a lot of time because of the complex structure of the LSTM model. The LSTM model also has some optimization algorithms [13], but they are not used in this study. Third, the time series model primarily serves as a tool for short-term forecasting, with its accuracy decreasing in the context of long-term predictions. It is crucial to acknowledge that for unexpected and abrupt 'black swan' events, time series models may not ensure precise forecasts, given their reliance on historical data analysis for future projections. Consequently, regular data updates are required to optimize the model's performance. Finally, no theoretical guidance can be adopted to identify the optimum number of hidden units, feedback delays and other key parameters during the establishment of ANN models [26]^.^, Determining the optimal model may necessitate substantial trial-and-error, thereby rendering the modeling process intricate and laborious.

## Conclusions

The overall incidence trend of gonorrhea in mainland China has been on the decline since 2004, with some periods exhibiting an upward trend. The incidence of gonorrhea displays a seasonal distribution, typically peaking in July and December each year. The SARIMA model, LSTM model, and SARIMA-LSTM model can all fit the monthly incidence time series data of gonorrhea in mainland China. However, in terms of predictive performance, the SARIMA-LSTM model outperforms the SARIMA and LSTM models, with the LSTM model surpassing the SARIMA model. This suggests that the SARIMA-LSTM model can serve as a preferred tool for time series analysis, providing evidence for the government to predict trends in gonorrhea incidence. The model's predictions indicate that the incidence of gonorrhea in mainland China will remain at a high level in 2024, necessitating that policymakers implement public health measures in advance to prevent the spread of the disease.

### Supplementary Information

**Additional file 1.**

## Data Availability

The dataset collected for this study can be retrieved from the subsequent web portal: http://www.nhc.gov.cn/jkj/new_index.shtml.
